# Entropy, Amnesia, and Abnormal Déjà Experiences

**DOI:** 10.3389/fpsyg.2022.794683

**Published:** 2022-07-27

**Authors:** Lana Frankle

**Affiliations:** School of Biomedical Sciences, College of Arts and Sciences, Kent State University, Kent, OH, United States

**Keywords:** entropy, amnesia, déjà vu, time distortion, déjà vécu

## Abstract

Previous research has contrasted fleeting erroneous experiences of familiarity with equally convincing, and often more stubborn erroneous experiences of remembering. While a subset of the former category may present as nonpathological “déjà vu,” the latter, termed “déjà vécu” can categorize a delusion-like confabulatory phenomenon first described in elderly dementia patients. Leading explanations for this experience include the dual process view, in which erroneous familiarity and erroneous recollection are elicited by inappropriate activation of the parahippocampal cortex and the hippocampus, respectively, and the more popular encoding-as-retrieval explanation in which normal memory encoding processes are falsely flagged and interpreted as memory retrieval. This paper presents a novel understanding of this recollective confabulation that builds on the encoding-as-retrieval hypothesis but more adequately accounts for the co-occurrence of persistent déjà vécu with both perceptual novelty and memory impairment, the latter of which occurs not only in progressive dementia but also in transient epileptic amnesia (TEA) and psychosis. It makes use of the growing interdisciplinary understanding of the fluidity of time and posits that the functioning of memory and the perception of novelty, long known to influence the subjective experience of time, may have a more fundamental effect on the flow of time.

## Introduction - Déjà Vécu: Current Knowledge and Presentations

Before delving into the association between déjà experiences and amnesia and possible explanations, it is important to clarify what is meant by abnormal déjà experiences and provide background and context. Between 60 and 80 percent of people have occasionally experienced déjà vu (Brown, 2003), making the occasional experience of déjà vu commonplace, and in this form it has been shown to be largely unrelated to psychopathology (Brown, 2003). But for some people, the passing sensation can extend into minutes, hours, days, or longer. When this occurs it can be described as disconcerting, if not distressing (Wells et al., 2014). This has alternately been described as “persistent déjà vu” and “déjà vécu” although a distinction can be drawn between a mere extension and protraction the normal déjà vu experience and the phenomenon of déjà vécu: the latter entails the sensation of remembering the events currently happening, and not just that they are familiar. Some researchers including Moulin (2018) further contrast persistent déjà vécu from recollective confabulation, suggesting the awareness of the recognition being false is what separates déjà vécu from recollective confabulation.

Pathological déjà vécu and recollective confabulation are rare, and while the prevalence is difficult to estimate, the number of studies and case reports documenting its occurrence is limited and largely restricted to a few discrete populations including elderly dementia patients (Moulin et al., 2005) in which it was first described, and temporal lobe epileptics (Illman et al., 2012). Patients who experience this often complain about events repeating themselves—they not only *feel* like things have happened before, but actually believe that they have. They will alter their behavior to avoid watching television programs they claim to have already seen or reading newspapers they have already read. One patient claimed to have already been interviewed by the BBC radio host interviewing him and to have already attended the funeral for a close friend who had passed (Moulin et al., 2005).

A notable feature of the case of this patient, AKP, as well as another documented patient, MA, was that the events that most often triggered their déjà vécu were particularly salient—the death of his friend in AKP’s case, and the Bali bombing in MA’s case were highlighted in their case study—whereas day-to-day activities like eating and personal hygiene did not elicit déjà vécu for either patient. Novel events being a trigger for the experience of déjà vécu or acting to magnify the sensation was also reported in several other patients (O'Connor et al., 2010) and this observation was further solidified with the use of a widely used word recollection task, in which false positives (words that had not been presented but were judged to have been) were more common among low-frequency (distinctive) words, like “polka” than high-frequency (common) words, like “mouse”—which is opposite to the expected pattern. Both the patients in this study also had prominent dementia, which by itself typically elicits significant errors in forced recall tasks. However, even dementia patients will typically show what is known as a mirror pattern word frequency effect (WFE) with this task, in which low frequency words show both a higher hit rate and a lower false alarm rate than high-frequency words (Balota et al., 2002) that is also seen in healthy individuals (Malmberg and Nelson, 2003). What is striking about this task in déjà vécu patients is the absence of this pattern. O'Connor et al. (2010) also used a “reverse Deese-Roediger-McDermott” task, a variation of the Deese-Roediger-McDermott task, in which subjects are presented with a list of words and then asked later if they remember the words, as well as another word in the same category that was not actually presented. Healthy participants will often falsely remember a related word (like “needle” if the list contains thread, haystack, cotton, and pin). But in O’Connor’s variation of the task, déjà vécu patients will also often falsely remember an unrelated lure word, like “Christmas” if the list contains red, green, blue, and black.

The association between false recollection experiences and novelty in déjà vécu patients proved to be more than random coincidence and can be judged to represent a critical feature of the syndrome (O'Connor and Moulin, 2010). One proposed explanation is that chronic déjà vécu patients experience constant or near-constant inappropriate remembering, but that this sensation is more noticeable to both them and their caregivers when they are experiencing something new (O'Connor et al., 2010). Another more compelling model has also been proposed in which novelty is more likely to elicit the subjective experience of memory due to a feature of the encoding-as-retrieval hypothesis in which hippocampal theta oscillations meant to signal memory encoding are inappropriately linked to downstream prefrontal regions and are processed as signals associated with retrieval (O'Connor et al., 2010). Whether the dysfunctional firing rates are present mainly in the CA1 hippocampal regions, the downstream/prefrontal regions, or both is still unestablished. My proposal accepts this explanation but goes beyond the existing discussion and examines the disruptions from normal theta and gamma oscillation patterns as an instance of divergence from brain criticality.

## Time as a Quale

Our day-to-day experience of time may seem like one of the few bedrock certainties of reality, but ample evidence exists that it is, on some level, a mental construct. As early as 1979, a paper by famed neuroscientist Benjamin Libet (Libet et al., 1979) demonstrated that while the average evoked response (AER) in the somatosensory cortex peaks just 100 ms following skin stimulation of the hand, neuronal adequacy for conscious awareness occurs only after a delay of approximately 500 ms after the original stimulation. Intriguingly, the time that subjects report becoming aware of the stimulus coincides with the initial evoked potential, and not with the later neural signature previously determined to represent conscious awareness. This phenomenon, termed “subjective referral” or “antedating” demonstrates not only that there exists a clear disconnect between time as we experience it and time as measured externally, but also the way in which unconscious mechanisms hide this disconnect to allow for more seamless interaction with the world. These groundbreaking results demonstrate that the translation of sensory input into awareness is not a direct and flawless process and is subject to manipulation. In later decades, psychophysics researchers outlined and quantified psychological factors that impact time dilation, compression, and the subjective flow of time—namely, factors like attention to time and arousal, influenced by the degree of engagement with the activity or perception at hand (Glicksohn, 2001; Kent et al., 2019). A relevant current example of particularly salient time dilations involves the months following the initial COVID-19 lockdown and quarantine in many countries in the spring of 2020. For many people who were not essential workers, their days abruptly became empty—virtually void of temporal landmarks, which led to various memory distortions including losing track of time and simultaneous over and under-estimation of how much time had passed (Grondin et al., 2020).

While psychophysics experiments show that different levels of engagement and individual differences affect subjective time perception, Eagleman and Pariyadath (2009) go one step further, saying that time itself is a quale—a fundamentally indescribable subjective sensation or perception, the important feature of which is that it is defined completely with respect to the person or other being who experiences it. Typical examples of qualia include things like color, which exists not because light is capable of taking on different wavelengths, but because people perceive those wavelengths in specific ways. They refer to a 2003 paper by Crick and Koch (2003), which also uses the less conventional example of motion perception as a quale, with the brain taking a series of closely spaced snapshots and piecing them together, only capable of perceiving one instant at a time, in which motion is “painted on” using changes between successive snapshots. The visual systems underlying these explicit representations, these qualia, are each susceptible to damage: using the examples already listed, color perception is lost in achromatopsia and motion perception is lost in akinetopsia. In akinetopsia, patients report seeing a series of snapshots that change, like a movie with still frames advancing too slowly, rather than painting on seamless movements the way most people take for granted. Eagleman and Pariyadath acknowledge their conjecture that time duration is similarly painted on is unproven. However, it is consistent with radical new conceptions of time suggested by developing branches of physics including the QBist notion of time evolution as a function of individual judgment (Barzegar, 2020). This is a necessary corollary of the QBist understanding of both quantum and classical events as observer-dependent. It would follow from this perspective and the fact that the outcomes of any measurements, quantum or otherwise, are contingent on a measurement having taken place at all; that the development of an observer’s future relies on their active observations and the frequency at which such observations take place (Barzegar, 2020).

Eagleman and Pariyadath (2009) showed that presenting oddball/unpredictable stimuli leads to time dilation. An even more compelling example of this type of manipulation comes from a recent study by Bechlivanidis et al. (2022) in which participants observe a simple visual sequence of simulated events (a colored block A shown on a screen hits another block, B, which causes it to hit a third, C). However, they altered the timescale of this representation such that in some trials, block C actually begins moving on the screen after the collision of A with B, but prior not only to the collision of B with C but also to the initial movement of B, by 150 ms. Participants will alter their subsequent reporting of the events as well as their within-trial estimates to align with a sequence that makes causal sense (that B hits C before C starts moving; Bechlivanidis et al., 2022). In yet another study, by Zheng et al. (2022) used to gage both time distinction and scene recognition in events shown depicted by hard, soft, or no boundaries (changes in the type of scene shown), it was demonstrated that participants were significantly more accurate at telling which scene was shown first if the two were separated by a soft boundary or no boundary (so as to appear as part of the same clip) than when separated by a hard boundary (appearing as scenes from unrelated film clips). In this study, recognition of target frames as old rather than a foil (frame not shown previously) did not depend on the type of boundary, but accuracy was increased for frames sooner after a (hard or soft) boundary (Zheng et al., 2022). The authors use these results as evidence of the way semantic visual boundaries can be used to enhance visual scene processing and episodic memory formation.

If time is a quale that is painted on like motion or color, it could be subject to similar disruptions in conscious perception, not only by manipulating image presentation in psychology experiments, but also due to genuine malfunction in clinical cases. For instance, the dramatic finding of subjective referral documented by Libet may have exceptions, and these exceptions manifest not in cases of supreme clarity or focused willpower but in severe psychiatric disorders, where they may present as delusions of control. Delusions of control, as well as subjective loss of feeling in control of one’s actions without the presence of full-blown delusion, which can both be unnerving or even subjectively terrifying aspects of schizophrenia spectrum disorder cases, have been hypothesized to relate to differences in this automatic antedating process (Blakemore et al., 2002).

One theory propounded by physicist Carlo Rovelli (Rovelli, 2020), centers around the progression of time and the arrow of time as defined by increasing entropy, which is contingent on the observables in a physical system, as depending on the factors that an observer of the system is coupled to. Notably, Rovelli does not claim there *are* any entropy differences between actual observers of physical systems, but rather demonstrates the potential for hypothetical subsystems within the universe to have differing arrows of time that depend on the unique way they interface with the universe. This interface is the coarse-graining by which the microstate of a system, which includes every possible piece of information about the system, is translated into a macrostate of relevant observable descriptors. Every conscious entity is a subsystem that is coupled to the rest of the universe *via* a set of macroscopic variables and the factors that determine which variables are relevant to a given observer are not random and confer an evolutionary advantage, which he described as “relevant relative information” (Rovelli, 2020). Support for this understanding comes from the intriguing observation by Alfonso Delgado-Bonal and Javier Martín-Torres that the wavelengths of light visible to humans and the majority of other seeing animals fall within a range that maximizes not the energy of wavelengths but the overlap between the maximal energy and the maximal entropy of wavelengths (a range of 500–555 nm), thereby maximizing the information efficiency (Delgado-Bonal and Martín-Torres, 2016).

Clear parallels exist here between Rovelli’s relational interpretation and QBism (Pienaar, 2021) with a major point of divergence being that QBism allows for the influence of observer-specific factors beyond the observables to which they have access as influencing time progression, and the conjecture that it makes sense to talk about such differences in human observers (Pienaar, 2021). This manuscript raises a specific example of an instance in which such observables (and hence active measurements) could differ between individuals, and argues that the consequences of this difference present as differences in the flow of time between people.

In gathering evidence for this conjecture, this paper draw from a range of academic sources showing perturbations of various measurements of entropy and their associations with each other and with markers of disease states and altered mental states. To be clear, these sources do use differing definitions of entropy: section four focuses on measures specifically designed for brain entropy, specifically electrodynamic oscillation, neural rhythms, and self-organized criticality; section five broadens to include basic thermodynamic measurements; and section six concerns information, or Shannon entropy. The entropic processes underlying the psychological arrow of time are brought in for comparison at each point by relaying the experiences associated with fluctuations in each type, particularly as they relate to perception, memory, and the experience of remembering. These definitions are not interchangeable as they all rely on unique systems of equations that define them—however, their shared terminology stems from the fact that they all represent a similar quantity. Changes in brain entropy, thermodynamic entropy, and information entropy can all be described as moving in the same direction in the same system, with the most relevant descriptor depending on how the system is being analyzed (Collell and Fauquet, 2015; Zimmern, 2020).

## Repetition Suppression, Attribute Amnesia, and Time Compression

The same 2009 study of Eagleman and Pariyadath (2009) speculated that the apparent time dilation in response to novelty that they documented may itself be relative—that the primary distortion of time is in fact a compression in response to the repetitive stimuli surrounding the oddball. They support this claim by drawing on the known phenomenon of repetition suppression, in which neuronal firing in higher cortical areas is suppressed in response to repeated presentation of the same stimuli, a mechanism for conserving neural resources. This diminished response occurs in response to stimuli that are predicted and is here suggested to underlie a subjective “time shrinking.”

Repetition suppression describes a neural phenomenon, but there are quantitative cognitive-level effects that show reduced attention being paid to repetitive stimuli. An effect in which people forget even salient features of a stimulus immediately after being exposed to it when not primed in advance to remember that attribute has aptly been termed attribute amnesia. The effect is greatly reduced with novel stimuli and much more pronounced for familiar or repeated stimuli (Chen and Howe, 2017). In these experiments, healthy adult participants were shown images containing objects and animals and asked unrelated filler questions. After over a 100 slides they were shown images containing animals, and then asked about the type of animal. If the animal shown had not been shown previously, subjects performed significantly better in their recall accuracy. The reason for this discrepancy that was proposed by the authors is that the subjects are able to remember what they have seen either way but have trouble recalling where exactly they have seen specific items or attributes. However, if a stimulus is novel, observers can identify the target because it is the only one they have seen. In other words, there is a natural coarse-graining of visual data that has been presented as it is categorized by the brain that influences the precision of either the perception or the immediately accessible storage of subsequent visual information.

Both the Eagleman and Pariyadath study that looks at repetition suppression and the Chen and Howe study that examines attribute amnesia take data from healthy volunteers and cannot be directly extrapolated to amnestic patients. However, there is evidence that clinical amnesia results from impairment in generating and binding the relevant perceptual details necessary to reconstruct episodic memories (Rosenbaum et al., 2009). This was demonstrated by significant lack of detail in self-generated narratives created by a patient with retrograde and anterograde amnesia—because these narratives are fictionalized and imagined, they did not rely directly on memories of the patient, and poor performance on this task signifies that the detail binding and scene construction process is deficient. An functional MRI (fMRI) study (Mullally et al., 2012) also showed scene construction deficits in an amnestic patient, and another fMRI study in healthy people show largely overlapping brain activation patterns during episodic memory recall and imagined scene construction (Hassabis et al., 2007). If detail binding and scene construction deficits underlie some common types of clinical memory loss (Hassabis et al., 2007; Rosenbaum et al., 2009; Mullally et al., 2012), they could also impact the way incoming sensory information is processed (Ding et al., 2017), similar to how disruption in the binding of visual details can lead to attribute amnesia in healthy subjects after viewing repetitive imagery. Indeed, binding between discrete features, such as object and location, was noted to be a key source of impairment in mixed-etiology amnesics (Chalfonte, 1996). Corroborating this link, visual attention deficits, specifically those corresponding to decreased neural activation in fMRI studies, have been observed in Alzheimer’s (Hao et al., 2005) and Lewy Body Disease (Revie et al., 2020) and have been linked to perceptual encoding deficits (Revie et al., 2020) that mirror detail binding problems in healthy people after prolonged stimulus repetition.

Another study, published in Nature Neuroscience, used an artificial neural network to model human time perception more accurately—that is, including the tendency to under-estimate and over-estimate time intervals in the same way as people. This network was comprised of connected hierarchical layers of nodes, each of which were attuned to a different level of feature detection, with the lower layers able to detect edges and contours and higher layers able to detect object-like archetypes (Roseboom et al., 2019). The time estimation given by each layer was a function of the number of changes it was able to detect, with an overall time estimation given by the model using a regression combining these estimates from all layers. Layers detecting more basic features tabulated more frequent changes, and faster passage of time. Time estimations at each level were determined by the threshold for changes to be detected, which the authors mention has parallels to the level of attention being paid by human observers when they estimate how much time has passed—the more attention an observer is paying to their visual surroundings, the more changes they will detect and the more time they will estimate having passed (Roseboom et al., 2019). An implication of this model is that people who observe fewer changes in their visual environment, such as due to limitations in their own visual processing ability, may feel time as passing faster.

While it was the repetition that caused the suppressed neural activation and consequent time compression, it was novel stimuli against the *backdrop* of repetition that led to relative time dilation (Pariyadath and Eagleman, 2007). The occurrence of the non-repeating stimulus acted as a prediction violation, which led to the observed time expansion. Further evidence of this relationship comes from another study that showed that positive prediction errors (numbers on a screen signaling monetary gain) bias time perception toward over-estimation (Toren et al., 2020). The authors explain this as evidence of the shared function of striatal dopaminergic neurons in prediction error-signaling and time estimation, as both of these functions were already localized to the striatum. This highlights the relevance of prediction error that occurs in déjà vécu patients in response to novel experiences, in the context of amnesia in which déjà vécu often occurs.

In summary, detail binding is responsible for delineating specific features between self-contained events (Ding et al., 2017) and impairment in this binding is featured in mixed-etiology amnesia (Chalfonte, 1996) and time compression. However, novel events may be able to be bypass some of these perceptual processing and binding deficits for the same reason novel stimuli did not trigger the same neural firing rate suppression as surrounding repeated visual stimuli in the study of Pariyadath and Eagleman (2007), or lead to the same cognitive-level attribute amnesia as the surrounding repeated stimuli in the study Chen and Howe (2017). That is, if a stimulus is new enough or different enough, it is easier to recall that its attributes were not actually associated with a different stimulus in an earlier visual presentation.

Patients with Alzheimer’s dementia (Güntekin et al., 2008), Parkinson’s dementia (Güntekin et al., 2020), vascular dementia (Jiang et al., 2017), and frontotemporal dementia (Hughes and Rowe, 2013), all show reduced response to visual and auditory oddball paradigms, meaning amnestic patients generally may not see the effect described in response to novel perceptions, despite the backdrop of binding deficits.^1^ But the high rates of false-alarm errors in the forced choice word recognition task to low-frequency, and not just high-frequency, words, as well as the high rates of false recall of semantically unrelated lure words in a reverse Deese-Roediger-McDermott task diverge markedly from the types of memory errors that occur in typical dementia or amnesia patients. O'Connor et al. (2010) use this atypical testing pattern to establish a partial basis for the role of novelty as a trigger in déjà vécu, also suggesting that novel or oddball stimuli are processed differently in the chronic déjà vécu subpopulation of memory loss patients.

Intriguingly, prediction errors of intermediate sensory priors/learning cues are also abnormally weighted in schizophrenia, another condition in which déjà vécu can present, which possibly acts to facilitate the development of both hallucinations and delusions (Millard et al., 2022). Lastly, prediction errors increase in the transition to seizures in the electroencephalograms (EEGs) of epileptic patients, the third group linked to abnormal déjà experiences and déjà vécu, and drop during the seizures themselves, most likely as a result of the strong coherence and regularity of electrical signals during synchronous firing (Principe et al., 2019). The authors identified the intersection of the CA1 and CA3 hippocampal subregions as having the best single correlation in prediction error matrices prior to seizures. The relevance of those regions will become clear in the following section.

## Prefrontal-Hippocampal Theta and Gamma Oscillations, and Criticality

O’Connor’s encoding-experienced-as-retrieval model links three standard assumptions about the dynamics of memory in healthy brains and speculates about how this could go awry in déjà vécu patients, explaining that:

1. theta oscillations in the hippocampus can signify either encoding or retrieval, but;
2. the intensity of these oscillations determines whether they are coupled to responding theta oscillations in the prefrontal cortex; and
3. the prefrontal cortex interprets them as a signal of retrieval rather than encoding.

Further coupling to theta oscillations in downstream prefrontal regions segments it as recollection (O'Connor et al., 2010; Kaplan et al., 2014; Tamura et al., 2017). Aberrant theta oscillation signaling of these downstream prefrontal neurons causes false sensation of recollection. Research has found distinct oscillation patterns in the hippocampus associated with encoding vs. retrieval, with

- fast gamma (60–100 Hz) increasing during memory formation (thought to facilitate connectivity between CA1 and the entorhinal cortex and to underlie information and scene processing), and
- slow gamma (30–45 Hz) increasing during memory retrieval (thought to facilitate connectivity between the CA3 and CA1 regions, underlying pattern completion; Colgin et al., 2009; Colgin, 2015).

One of the few studies to quantify EEG measurements of the déjà vu experience itself used bilateral medial temporal lobe (MTL) electrode stimulation in epileptic volunteers. It found significantly increased correlation in theta rhythms coupling the rhinal cortex (of which the entorhinal cortex mentioned previously is a part) and the hippocampus following stimulations that triggered a déjà vu response vs. those that did not (Bartolomei et al., 2012), which the authors discuss as further evidence of the experience of both déjà vu and déjà vécu as being due to increased correlations between MTL structures, and more complicated than a single localizable dysfunction. A meta-review of electrical brain stimulation since Penfield first developed the technique also found the rhinal cortex to be the region most likely to induce reminiscence (vague or detailed episodic memories of an experience in the patient’s past) when stimulated, leading to its description as a gatekeeper for declarative memories (Fernández and Tendolkar, 2006; Curot et al., 2017). In normal waking experience, it carries the semantic sensory representations that comprise experiences that can be encoded or trigger a recollection (Fernández and Tendolkar, 2006).

In healthy brains, activation of the dentate gyrus, CA3, entorhinal cortex, and CA1 are involved in both novelty processing and recognition memory (Lee et al., 2020). Sensory signals from the entorhinal cortex project onto the dentate gyrus and then the CA3 hippocampal subregion which mediate pattern separation and previously stored memory streams, before projecting onto the CA1 hippocampal subregion (Stepan et al., 2015), where firing indicates either novelty or recollection in O’Connor’s model.

Because these regions, particularly the dentate gyrus and CA3, are involved in both novelty and recognition, damage or dysfunction in these areas may impact recognition memory and novelty detection. Dementia patients who have trouble with scene recognition and associative object memory also have a reduced neural response to novelty of these same image types (Bastin et al., 2019). Due to its established role in pattern separation, the dentate gyrus and CA3 are proposed to distinguish closely related sequences of imagery as representing distinct memories (Lee et al., 2020)—a function closely tied to the detail binding functions described above that are relevant to attribute amnesia and repetition suppression and are impaired in dementia models. The item and scene discrimination functions of these regions make them necessary for spatial memory, but also for the awareness of a scene’s novelty as they hold the expectations against which the sensory stream from the entorhinal cortex is compared. Dvorak et al. (2021) describe similar findings and speculate that dorsal spiking from the dentate gyrus facilitates information transfer from the lateral entorhinal cortex into Ammon’s horn, a region that includes CA2 and CA3, which primes the CA3 region to be preferentially activated by mental and emotional contextual cues during retrieval. They add that this dorsal spiking acts to couple slow gamma waves between the dentate gyrus and CA1 maximizing information transfer efficiency between brain regions.

There are different levels to pattern separation, and in an fMRI study of healthy participants with different APOE genotypes, false responses of “repeat” on a spatial object recognition test were attributed to a lack of relational, rather than item-based pattern recognition, dependent on CA3 rather than the dentate gyrus (Lee et al., 2020). In trials resulting in this type of error, item recognition was intact but the memory of the item’s associated source was faulty—the item is recognized, but the spatial location is not tagged as being new, leading to CA1 activation and oscillation, so the participant falsely reports it as a repeat. If a related mechanism is responsible for persistent and pathological false recognition experiences in persistent déjà vécu, it would be fully compatible with the lack of pattern separation and details binding that underlie the subtle time compression effects in diminished episodic memory. This model of clinical déjà phenomena would fit the parallels drawn to non-clinical attribute amnesia, repetition suppression, and time compression.

Spatial memory retrieval in humans is known to cue increases in theta and gamma band oscillation from the MTL (Kaplan et al., 2014), which contains the hippocampus, parahippocampal cortex, entorhinal cortex, and perirhinal cortex. It is further known that these oscillations become coupled with prefrontal cortical oscillations of the same frequency. Researchers have termed increases in synchronization within a certain frequency, such as theta or gamma *long-range temporal correlation* or LRTC (Palva and Palva, 2018; Zimmern, 2020). LRTC is a neural correlate of long-term memory, but actually represents a geometrically propagating perturbation from critical or critical-like states. Criticality is a property of physical systems existing at a point of phase transition, and self-organized criticality, as occurs in biological systems such as the brain, as well as many other natural phenomena, refers to the tendency of these systems to have a point of spontaneous convergence, or critical point, in this instance measured by the synchrony of electric propagation. Brain criticality exists along the edge between order and disorder. Divergence toward increased criticality, or supercritical states, occur in times of increased firing, such as an epileptic seizure, with a lack of neural firing being a divergence toward hypocriticality, also not optimal (Zimmern, 2020). Marked divergences occur in extreme and disease states, like epilepsy, coma, or anesthesia, as well as drug use. Fluctuations within normal range also occur regularly in healthy brains and are self-correcting back toward baseline criticality levels. Several studies have shown changes to self-organized criticality and its typical oscillations and fluctuations in dementia (Stam et al., 2005; Montez et al., 2009; Vysata et al., 2014). Most interestingly, divergence from criticality can be measured in terms of entropy—and it has an effect on the perception of time. This idea will be explored further below.

Neuronal oscillations are known to be entrained by sensory perception, such as the rhythm induced by perception of most languages’ speech as existing at a frequency at or close to 5 Hz (Palva and Palva, 2018). However, more pertinent to this analysis, there is also ample evidence that neuronal oscillations in turn entrain perceptual cycles, particularly with regard to temporal perception and associated duration estimations (VanRullen, 2016). Differences in neuronal firing frequency rates prior to stimulus presentation are described to have an influence not on perceptual accuracy but on the rate of stimulus perception, sometimes termed “frame rate” analogous to a film reel or video clip (VanRullen, 2016). Within alpha rhythms measured, peak alpha rhythms corresponded to increased temporal resolution in visual simultaneity judgments, with decreased alpha rhythms corresponding to poorer temporal resolution in these judgments. Their approach focused on the effect of brain wave frequency, as well as the phase of each oscillation (rather than directly measuring or controlling for the degree of criticality), on judgments of temporal accuracy. Other authors have linked the frequency of brain oscillations directly to both entropy and temporal judgments, with slower oscillations and higher frequencies both leading to temporal expansion (Déli and Kisvárday, 2020).

A recent paper (Kragel et al., 2021) showed convincing evidence, using measurements from internal electrodes in epileptic patients, of both entrainment of perceptual cycles by hippocampal theta oscillations and the instigation of these hippocampal theta oscillations by semantic features of visual scene processing. This study tracked eye movements and correlated them with intracranial EEG (iEEG) measurements to determine temporally precise correlations that occur during perceptual encoding, and distinguished between retrieval sequences that occur at the beginning of image processing and encoding sequences that occur subsequently, to identify neural and eye movement signatures of revisitation, or redirection of eye movements to previously processed semantic components of an image during the initial encoding session. The authors focus on the fact that hippocampal theta oscillations occur pre-revisitation, potentially driving the eye movements that characterize it. However, they also note that hippocampal theta oscillations are significantly increased post-revisitation, meaning the eye movements that comprise this revisitation may increase hippocampal theta oscillations in turn—which would also be consistent with known effects of saccadic eye movements on time perception (Morrone et al., 2005; Yu et al., 2017). This study was conducted using epileptic patients, but the reason for this selection was the use of internally placed electrodes, and the results presented were generalized to the population at large. This paper did not mention recollective confabulation or déjà vu, however, it does provide additional support for perceptual and electroencephalographic rhythm interplays and cross-modulation during the presentation of novel visual stimuli.

Supercriticality, as that associated with the use of psychedelic drugs, is associated with an increase in entropy (Carhart-Harris et al., 2014; Varley et al., 2020) whereas subcriticality, associated with the use of depressant drugs, among other conditions (Zimmern, 2020), is associated with lower entropy (Varley et al., 2020). The LRTC, representing temporary increases in criticality, that are hallmarks of memory retrieval can in other contexts be associated with attentional impairments and worse performance on tasks requiring sustained attention (Simola et al., 2017). False recollection experiences described in previous sections would, if represented by similar hippocampal-prefrontal oscillations to that of normal recollection as speculated by O'Connor et al. (2010) be represented by LRTC, and skew toward supercriticality, increased macro-scale entropy (Cocchi et al., 2017), and a slower “frame rate” of perceived time (VanRullen, 2016; Déli and Kisvárday, 2020). Decay of these LRTC autocorrelations and return to the baseline critical-like state, however, would involve an entropy fluctuation back in the opposite direction. Such a pattern would mirror the proposed brain entropy fluctuations occurring from use of LSD, in which lasting therapeutic effects of the drug result from entering a malleable high-entropy brain state while under the acute influence of LSD and then returning to a subsequent baseline state lower than the initial baseline state (Carhart-Harris and Friston, 2019). These details are relevant for reasons that will be made clear in subsequent sections, but ultimately pertain to the causal relationship between entropy and time.

## Known Brain Entropy Fluctuations and Time: Sleep, Saccades, and Temperature

While the role of entropy fluctuations and time in cases of chronic déjà vécu may be largely unexplored, perturbations of brain entropy having a subjective effect on temporal processing are not undocumented or unsupported. The second law of thermodynamics, one of the most well-known scientific principles to many laypeople, dictates that entropy must always increase. However, this law applies only to isolated systems—for systems that are not isolated, the law is true on a probabilistic level, but not as an absolute. Systems that are not isolated often violate entropy increase—and they do so without violating any physical laws. A common example would be a household refrigerator, which decreases the temperature of a specific area of space that is still surrounded by room-temperature air. It requires energy to do this, heating the air around it that is not contained in the refrigerated space. To avoid violating the second law, the entropy increase (heat created) must be greater than the entropy decrease it causes by cooling the space inside of it. The difference between the entropy increase and the entropy decrease is referred to as the *efficiency* of the refrigerator, and no refrigerator, no matter how meticulously crafted, can ever be 100% efficient (at least in this sense; Donev et al., 2021).

However, the accumulation of entropy is also inextricably linked with the progression of time—a fact that is particularly salient because no other physical law shares this property. The directionality of time is obvious to everyone and is taken for granted, but laws of physics are what is called time-reversible, meaning that all physical laws, from descriptions of force interacting with macroscopic objects to principles governing the spin of electrons, work equally well in both directions of time—with the exception of the second law of thermodynamics. The increase of entropy will always align with the flow of time from past to future. In fact, one could just as easily say that time must flow in the direction of entropy increase as that entropy must increase with time—many physicists would say it is more accurate to say that increasing entropy drives the so-called arrow of time, rather than the arrow of time driving entropy increase (Parrondo et al., 2009). Without some form of increasing entropy, the future is indistinguishable from the past, and whether a film clip is being played forward or in reverse cannot be gaged. To make this relevant to déjà vécu or psychology generally, there is also a *psychological arrow of time* in addition to the more well-known thermodynamic arrow. This psychological arrow refers to the direction of our memories marking the past and our expectations and plans regarding the future—you would not talk of remembering the future or plan to make changes to the past. Therefore, the psychological arrow of time is aligned with the thermodynamic arrow of time. This is not incidental, nor should it be taken for granted. A few papers (Maccone, 2009; Mlodinow and Brun, 2014; Hemmo and Shenker, 2019; Irwin et al., 2020) have speculated about why it seems that the psychological and thermodynamic arrows must always align, and there is no clear consensus. One assertion is that a change in the direction of the thermodynamic arrow of time of an individual’s environment would necessitate a corresponding change in the individual’s psychological arrow, that the two are intrinsically coupled (Mlodinow and Brun, 2014). One can speak not only of the arrow’s direction but also of its magnitude, for the case of both external as well as internal time (Ghaderi, 2019). Just as thermodynamic entropy of a physical system will increase, but may increase at a different rate depending on the specifics of the system itself (how concentrated a cluster of gas particles is within one corner of a box, the air pressure within the box, and other factors) the progression of time as perceived by an individual—their psychological arrow—may depend on factors like their boredom and level of attention, the factors that Glickson and Kent (Glicksohn, 2001; Kent et al., 2019) determined to affect estimations of the passage of time. Furthermore, because the brain is also a physical system, but is not an isolated system (it has contact with other systems and exchanges heat and energy with them) its entropy, unlike the entropy of the universe, can potentially either increase or decrease, corresponding to either a forward-feeding or reversed arrow of time (Ghaderi, 2019). Entropy rate is dependent on both temperature and heat transfer (enthalpy), as governed by the following formula:


dS=dQ/T

Where *S* is entropy, *Q* is enthalpy, and *T* is temperature. In the brain, the cortex generates and emits heat, which is dissipated by the surrounding cerebrospinal fluid (CSF; Ghaderi, 2019). Brain metabolism has been shown to be associated with subjective distortions of time (Eagleman, 2008). Overestimation of short time intervals was associated with higher global brain activity, and underestimation of time intervals was associated with lower global brain activity. Blood oxygen level as measured by regional cerebral blood flow (rCBF) signal has been shown to be decreased in non-oculomotor regions during saccade production (Paus et al., 1995), during which there is also time compression of visual perceptions (Morrone et al., 2005). The decreased blood flow parallels the decreased BOLD blood oxygenation levels observed in response to repeated visual stimuli (Pariyadath and Eagleman, 2012), as described in earlier sections.

Brains undergo a suspension of homeothermic regulation during sleep, or a drop in the overall temperature of the brain, meaning a potential drop in brain entropy during REM sleep as well. Of note, the eye movements that characterize REM sleep are saccade-like, and this drop in temperature is accompanied by a corresponding decrease in variational free energy and in prediction error (Hobson et al., 2014) a counterpart to the prediction error increases of déjà vécu. While the Hobson and Friston paper does not refer directly to entropy fluctuations, the famous free energy principle at work in their analysis depends on placing an upper bound on system entropy. And in their paper, the reduction in overall temperature corresponds to a reduction in prediction error, corresponding to entropy fluctuations, during sleep.^2^

These papers collectively set a general precedent for the existence of brain entropy fluctuations as well as their association with both brain activity levels and changes in time perception: with decreased brain activation being linked to shorter perceived duration and decreases in prediction errors and free energy being linked to decreases in brain temperature. The Ghaderi paper emphasizes the underpinning of the psychological arrow of time as grounded in the laws of physics and relativity. It describes the possibility for the external arrow of time to be at odds with the internal arrow of time. In the characterizations laid out in other parts of this paper, it would be more accurate to say there is no objective external arrow of time of any relevance, but changes in brain activity and brain entropy, in particular in déjà vécu cases, do have an effect on the magnitude, if not the direction, of time’s arrow. An even stronger link can be found in that lower body temperature was found in an Alzheimer’s model (Vandal et al., 2016) and linked to the disease progression, and thermal dysregulation is an often-noted characteristic of schizophrenic patients (Chong and Castle, 2004), who also have significant rates of abnormal time experiences (Blom et al., 2021) and déjà vécu (Stanghellini et al., 2016). Lastly, thermoregulation circuits are both affected by, and can play a role in, the development of seizures (Pollandt and Bleck, 2018).

## Neural Network Models of Recognition; Learning, Novelty, and Information

The previous two sections focus on brain entropy and divergence from criticality and brain entropy as part of a thermodynamic system. However, there are other distinct but largely overlapping definitions of entropy that also apply to brain entropy fluctuations and potentially to their role in déjà vécu experiences, including those provided by neural network models and information theory.

It is worth noting that the paper of Dvorak et al. (2021) demonstrates an effect of the slow gamma waves not only on information transfer rates but also on information transfer *efficiency*. Simply put, information transfer in this context is a measure of functional, effective connectivity between separate brain regions, of conveying information in the form of neuronal firing from a presynaptic source to a postsynaptic sink—such as between a source in the entorhinal cortex and a sink in the dentate gyrus, or alternatively between a source in the dentate gyrus and a sink in the CA1 region. But the terms source and sink also have more general meanings within information theory, encompassing any instance of information transfer between spatially separated points. This can also be measured in terms of *transfer entropy* (Ursino et al., 2020). Transfer entropy is a measurement related to mutual information, such as between connected brain regions, but measuring the raw transfer entropy of effective connectivity will include both meaningful information transfer and a noise component (Harris et al., 2019). Increasing the amount of information transferred is therefore slightly but crucially different from increasing the efficiency of this transfer, as increased information transfer efficiency can result from a minimization of this transfer entropy noise. This reduction in transfer entropy can be equated to a reduction in the information entropy of the neural processes at work. Dvorak’s paper examined the differences between the neural underpinnings of encoding and recollection, but did not attempt to explain any cases of conflation between the two. As discussed, deficiency in one link in the complex neural chain of information processing—such as the relational pattern separation function of the CA3 subregion—could cause miscategorization of novel experiences as recollected. And, while the Dvorak paper did not look at how this mechanism could go awry, the result they reported of the increased efficiency of the information transfer between the dentate gyrus and the CA1 region acted through the CA3 intermediary.

Notably, CA1 neurons are differentially sensitive to different inputs: similarly to primary visual cortex neurons having a maximal firing rate for a specific orientation of a line, and incrementally decreasing firing rates for less similar visual input orientations (Butts and Goldman, 2006), hippocampal place cells also exhibit a “tuning curve” in which they fire maximally in response to cues from a given location, but also, less strongly, to similar cues (Roy and Narayanan, 2021). The peak of a neuron’s tuning curve is where it fires most frequently, whereas the slope of a tuning curve can enhance discrimination more rapidly by enabling large changes in firing rate from small changes in stimulus (Butts and Goldman, 2006). For CA1 place cells receiving projections from the CA3 region, variability or noise in the signals decreased Shannon mutual information, which has a (negative) noise entropy term, and mutual information was greatest at the slope of this tuning curve (Roy and Narayanan, 2021). This study does not translate directly to humans or to false recollection; however, it would be interesting to observe the behavior of place cell tuning curves and mutual information and its associated entropy term, in response to spatial cues that are falsely familiar.

Other lines of research also provide indirect support for fluctuations of information entropy in cases of false recollection. For instance, the retrieval process has been modeled in neural networks as a classification of external stimuli. An input is passed to a first processing network and if it significantly overlaps with a prototype pattern it is then passed to a second network and recognized using the constraint that it belongs to the initial category established by the first network. However, if one breaks the connection between these networks, the first network will still recognize it as belonging to that class, but the second network will be overloaded with possible specific object or instance matches due to not being constrained by the information matching a single prototype within the first network (Gutfreund and Toulouse, 1992). In neural network models, this scenario is categorized by increased entropy and coarse graining of identification. This network dysfunction directly mirrors memory failure and visual agnosias seen in patients (Harris et al., 2019) and in fact itself characterizes a form of detail binding deficits within a neural network. This pattern of errors within this storage configuration in associative memory is associated with an increase in synaptic noise, equivalent to an increase in temperature (Gutfreund and Toulouse, 1992). This increase in temperature can be directly mapped onto an increase in the entropy of the model. In dementia and amnesia generally, deficits in associative memory are characterized by the same increase in entropy when measured in other ways, as described in previous sections. How would the introduction of novelty against this backdrop manifest in a neural network model consistent with the previous characterizations of déjà vécu?

In information theory models, less predictable, low-probability events contain higher self-information and higher entropy—leading to a decrease in the average entropy of subsequent information. (Kim, 2019) This pattern forms a direct parallel to the entropy increase as determined by neural oscillations and divergence from critical-like states that occurs during a recollection experience (and arguably also during a false recollection experience), followed by the decrease in entropy that occurs in tandem with the return to the baseline state of lower entropy and closer to criticality. There is also direct evidence of the effect of this pattern on time perception. Hansen et al. (2021) observe that final notes of musical phrases that are quantified as high-entropy and greater in uncertainty are experienced as lengthened. They posit this could be a perceptual bias of the listener resulting from the high demands of processing uncertainty.

## Discussion - The Role of Entropy and Altered Time in Clinical Déjà Experiences

So far we have examined how brain entropy fluctuations could underlie many qualities specific to the déjà vécu experience; how these fluctuations present on multiple levels in tandem: electromagnetic, thermodynamic, and informational; and how these tie into the experience of altered time and apparent repetition or recollection. But what does this actually mean in terms of providing an explanation for the subjective phenomenon? There is still a disconnect, given the above explanations, between visual sensory input and the feeling of retrieving a memory, which becomes complicated by a necessary, and unprecedented assumption that has not yet been stated directly: time itself is what is disrupted in these cases, meaning that piecing together the causal sequence is not a straightforward exercise. Take for example, a quote from patient AKP, upon being interviewed by the BBC,

*“The surroundings are the same, and that without being offensive your sight against the filing cabinets and so on, and the heater, it looks familiar. Since then, (my) memory got slightly worse, that’s all. Besides, you asked the same questions. Why I remember them, and whether they are really the same, I do not know, but it seems like it.”—*(Moulin, 2018)

If the QBist interpretation were to be applied here, time in these cases, when defined as the frame rate of visual experience, is given by the observables that an entity has access to perceiving, rather than the other way around. Therefore, just as other scholars describe increases in entropy as driving the progression of time, rather than entropy increasing *with time* (Parrondo et al., 2009), it also makes more sense to say that visual perception can cause disruptions in time, rather than them resulting entirely from an intrinsic factor in the brains of déjà vécu patients leading to a temporal switch and the sensation of new visual stimuli as being repeated. AKP perceiving the filing cabinets and the heater in the news studio in the above example, or another similar example in another patient, creates a kind of cascade (depicted in graphical format on Table 1):

**Table 1:** Dissecting the anatomy of a déjà vécu experience.

**Prior to event/stimulus**	**Novel/surprising event** **+ 300–400 ms post-event**	**500–600 ms post-event** **decay of autocorrelation**
(Low entropy) brain state	LRTC between hippocampus and PFC	Decay of LRTC
	False recollection	Temporarily more sub-critical, lower entropy brain state
	High information content	Fluctuation in subjective time
	Incr. entropy responding brain state	Conscious perception of event

The time lengths given in this table are based on the time lengths required for conscious processing (Herzog et al., 2016) and the range of time lengths for decay of LRTC (Zimmern, 2020). The experience of déjà vécu can be persistent or pervasive so as to seem like a continuous re-experiencing of events, however, even in these cases each new percept is associated with its own cycle described above.

1. Chronically decreased brain metabolism, brain heat, and brain entropy exist as the starting point in most déjà vécu cases that occur within the context of amnesia, including that of dementia, epileptic amnesia, and the few psychogenic cases that have been linked to psychosis.
2. Novel visual information leads to a temporary increase, followed by a returning decrease, in brain entropy levels, and does so by the following mechanism: incoming visual stimuli arrive to the CA1 hippocampal subregion via the entorhinal cortex, where they merge with input streams from the dentate gyrus and CA3 subregions, which compare previous visual memories in an item-based (dentate gyrus) and relational (CA3) manner to determine whether CA1 firing patterns will signal novelty or repetition. Deficits in CA3 signaling will leave intact correct first-order perception and item-based recognition, but result in an inability to recognize a different context, leading to false recognition and a CA1 firing pattern and generated neuronal oscillations characteristic thereof.
3. These oscillations create LRTC patterns, and an associated divergence from the critical-like state of the brain, leading to temporary supercritical firing patterns, or a temporary increase, then decrease, in brain criticality and entropy, which would also manifest on levels of temperature and information.
4. This fluctuation in brain entropy levels exists also as a fluctuation in the psychological arrow of time, an actual repetition of the moment in question. While the external visual stimulus, whatever it may be, is necessary to act as a trigger for this momentary reversal, the reversal, and repetition, is also genuine—the perception follows the false recollection (however trivially, as the time intervals at play are the timescales of sensory perceptions and initial sensory processing, that is, at most, hundreds of milliseconds). This conjecture follows directly from the brain entropy fluctuations already demonstrated existing as concrete measurable phenomena that determine rather than demarcate the flow of time, and the erosion of the always artificial boundaries between the individual psychological arrow of time and an objective external thermodynamic arrow of time.

While some parts of this paper delve into abstract concepts, it also features easily testable predictions, namely by applying temporal EEG to déjà vécu patients while undergoing some of the same tests, such as the forced-choice word recognition test and the reverse Deese-Roediger-McDermott test, that were first used on this population almost 2 decades ago. fMRI BOLD or rCBF or FDG-positron emission tomography (PET) studies during these testing conditions could provide metabolic measurements to supplement the EEG correlates of entropy, and brain and body temperature could also be easily measured in these patient groups.

## Data Availability Statement

The original contributions presented in the study are included in the article/supplementary material; further inquiries can be directed to the corresponding author.

## Author Contributions

LF conceived of the hypothesis and wrote the manuscript.

## Funding

This publication was made possible in part by support from the Kent State University Open Access Publishing Fund.

## Conflict of Interest

The author declares that the research was conducted in the absence of any commercial or financial relationships that could be construed as a potential conflict of interest.

## Publisher’s Note

All claims expressed in this article are solely those of the authors and do not necessarily represent those of their affiliated organizations, or those of the publisher, the editors and the reviewers. Any product that may be evaluated in this article, or claim that may be made by its manufacturer, is not guaranteed or endorsed by the publisher.

## Footnotes

^1^Notably, different types of dementia are also known to have different effects on prediction error signaling – patients with Alzheimer’s have deficits in following conversations in the presence of background noise, grouping and processing sounds over time, and processing melodic contour, all of which require successful implementation of auditory predictions, and dementia associated with Lewy Bodies Disease can cause complex visual hallucinations associated with abnormal weighting of intermediate sensory predictions. Patients with Alzheimer’s are still able to perform a weather prediction task requiring probabilistic learning on micro-timescales, but patients with Parkinson’s, Huntington’s, or frontotemporal dementias struggle with this task (Kocagoncu et al., 2021).

^2^While a complete account of deja vecu explained in terms of the free energy principle is beyond the scope of this paper, it would be consistent with the arguments proposed here as the coarse-graining of macroscopic observables also directly describes the level of detail of the recognition density of the posterior states to which the priors of the generative model are compared (Ramstead et al., 2020).
